# Relationship between financial speculation and food prices or price volatility: applying the principles of evidence-based medicine to current debates in Germany

**DOI:** 10.1186/1744-8603-9-44

**Published:** 2013-10-16

**Authors:** Kayvan Bozorgmehr, Sabine Gabrysch, Olaf Müller, Florian Neuhann, Irmgard Jordan, Michael Knipper, Oliver Razum

**Affiliations:** 1 Department of General Practice & Health Services Research, University of Heidelberg, Heidelberg, Germany; 2 Institute of Public Health, University of Heidelberg, Heidelberg, Germany; 3 Institute of Nutritional Sciences, Justus Liebig University Giessen, Giessen, Germany; 4 Institute of the History of Medicine, University Giessen, Giessen, Germany; 5 Department of Epidemiology & International Public Health, School of Public Health, Bielefeld University, D-33501 Bielefeld, Germany

**Keywords:** Financial speculation, Food prices, Price volatility, Food security, Systematic review, Critical appraisal, Global health

## Abstract

There is an unresolved debate about the potential effects of financial speculation on food prices and price volatility. Germany’s largest financial institution and leading global investment bank recently decided to continue investing in agricultural commodities, stating that there is little empirical evidence to support the notion that the growth of agricultural-based financial products has caused price increases or volatility. The statement is supported by a recently published literature review, which concludes that financial speculation does not have an adverse effect on the functioning of the agricultural commodities market. As public health professionals concerned with global food insecurity, we have appraised the methodological quality of the review using a validated and reliable appraisal tool. The appraisal revealed major shortcomings in the methodological quality of the review. These were particularly related to intransparencies in the search strategy and in the selection/presentation of studies and findings; the neglect of the possibility of publication bias; a lack of objective or rigorous criteria for assessing the scientific quality of included studies and for the formulation of conclusions. Based on the results of our appraisal, we conclude that it is not justified to reject the hypothesis that financial speculation might have adverse effects on food prices/price volatility. We hope to initiate reflections about scientific standards beyond the boundaries of disciplines and call for high quality, rigorous systematic reviews on the effects of financial speculation on food prices or price volatility.

## Background

Germany’s largest financial institution and leading global investment bank continues investing in agricultural commodities [1], stating that “there is little empirical evidence to support the notion that the growth of agricultural-based financial products has caused price increases or volatility” [2]. The bank’s statement is supported by a literature review recently published by researchers based in Germany at the University of Halle-Wittenberg and the 'Leibniz Institute for Agricultural Development in Central and Eastern Europe’. The review, which comprises 35 studies published between 2010 and 2012, concludes that “[..] financial speculation does *not* have an adverse effect on the functioning of the agricultural commodities market [..]. If one considers the empirical evidence in its entirety [..], the alarm raised by civil-society organizations must, inevitably, be regarded as *false* alarm.” [3]*(p.20, emphases in original).* The “alarm” refers to a campaign, launched by a broad coalition of civil society organisations (CSOs) in Germany, which advocate for regulation of investments in agricultural commodities, claiming that the increase in financial products has led to higher food prices and volatility of markets. International medical journals have recently been a platform for similar debates [4-6]. In Germany, this controversy is increasingly becoming a matter of “science vs. civil society”. In an open letter to the Federal President of Germany, the lead authors of the review [3], supported by 40 German scientists and agricultural economists, have called for a more differentiated discussion about the potential effects of financial speculations and actively advocated against the regulation of agricultural markets. Building their arguments mainly upon the cited review [3], they expressed their concern that arguments which are scientifically not supportable had been invoked by CSOs. Although these debates take place in a national context, any policy implications drawn from such conclusions might have global consequences.

## Discussion

As public health professionals concerned with global food insecurity, we have followed the increasingly controversial public debates and read the review [3] invoked by the scientists as proof of 'no evidence’ for a relationship between speculations and food prices or price volatility. However, we find this 'proof’ unconvincing, at least if the standards of evidence-based medicine (EBM) are applied. From an EBM perspective, the appropriate research design for a review that aims to draw the conclusion 'no evidence for an adverse effect of X on Y’ is the systematic review. The rigorous standards of the systematic review have been developed to make transparent how research findings are derived from a review of literature, while at the same time minimising bias caused by selective search strategies, selective inclusion or exclusion of literature, unsystematic extraction and presentation of results, or subjective appraisal of the quality or importance of research findings within the included literature.

We have applied these standards to the above-mentioned review [3] using AMSTAR, a structured, validated tool for appraising the methodological quality of reviews [7]. All authors independently read and appraised the review.

In line with the usual AMSTAR procedure, the mean (95% confidence interval, CI) of all independently established total scores of the appraisal was interpreted as reflecting the quality of the review in question. Mean inter-item correlations were calculated and inter-rater reliability regarding the absolute agreement on the total scores was assessed by the intraclass correlation (ICC) derived from a two-way random effects model [8] using SPSS 16.0.

The review was rated an average total score of 1.71 (95% CI: 0.83 - 2.59) in the AMSTAR appraisal out of a maximum achievable score of 11. Mean inter-item correlations were moderate (0.73) and inter-rater reliability on the total score was very high (ICC=0.94, 95% CI: 0.87 - 0.98).

**Figure 1:**
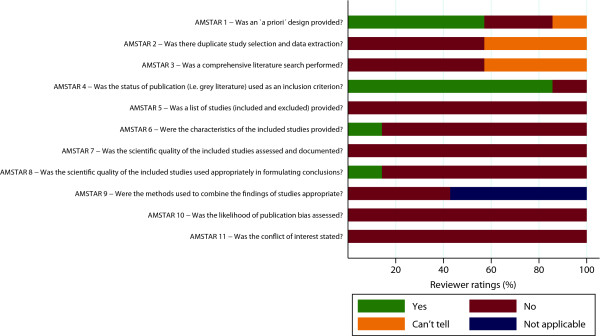
Ratings on each of the 11 items of the AMSTAR appraisal tool (N=7 raters).*Percentages refer to ratings of 7 raters who independently appraised Will et al. [3] using the AMSTAR appraisal tool.

Figure 1 illustrates how the review [3] scored in each single dimension of the appraisal tool. The largest discrepancy between the EBM standards and the review [3] were related to: intransparencies in the search strategy (AMSTAR3) and in the selection/presentation of studies and findings (AMSTAR2&5); the neglect of the possibility of publication bias (AMSTAR10); a lack of disclosure of potential conflicts of interest (AMSTAR11); and most notably a lack of objective or rigorous 'a priori’ criteria for assessing the scientific quality of included studies and for the formulation of conclusions (AMSTAR7-8) (Figure 1). Excluding indicators rated 'not applicable’ (which was the case only for AMSTAR9) did not change the mean total scores/ICC of the analysis.

Due to the fact that all initial assessments fed into the review without any post-hoc 'corrections’, it is principally possible that false positive or false negative judgements affected our results (see Additional file 1, p.3). However, as Figure 1 and the high ICC suggest, such minor discrepancies do not considerably affect the overall judgement on the quality of the review.

Not all AMSTAR indicators were easily transferable to the review [3] and members of the appraisal team had to make individual judgements. It is important to note, however, that the AMSTAR standards, which mainly refer to statistical tests, were not applied rigidly but rather flexibly in this context, with explicit considerations about the applicability of each item and the rationale behind (see Additional file 1, pp.1-2). Moreover, the high inter-rater reliability (ICC) shows that the AMSTAR tool can be meaningfully applied for assessing the methodological quality of a review regardless of its scientific discipline.

Informed by the principles of EBM and in an attempt to improve the methodological and reporting quality of economic reviews, similar standards have just recently been formulated in the field of economics by the 'Meta-Analysis of Economics Research Network’ (MAER) [9]. The rationale for using the AMSTAR guideline instead of the one tailored for the field of economics was that: (i) AMSTAR has been rigorously validated (which is not the case for many appraisal checklists, including the MAER guideline as far the authors can judge from the literature [9]); (ii) the MAER guideline does not provide an overall summary score (which makes it difficult to aggregate judgements about quality) and puts an emphasis on meta-analyses and pooling techniques (which, however, does not lessen its applicability to the review in question); (iii) despite the overlap of the MAER standards with some of the AMSTAR principles, we felt more comfortable using a 'lens’ from our own research background to make a judgement about a study from a different field.

## Conclusion

Given the obvious methodological shortcomings of the review [3] (Figure 1), we conclude that it is not justified to reject the hypothesis that financial speculation has adverse effects on food prices/price volatility. If 'financial speculation’ was a drug, and 'rising food prices’ or 'price volatility’ its potential adverse effect, the current 'proof’ [3] would be insufficient to falsify critical assertions about these effects when the standards of our discipline [7] are applied. These standards [7,9] are also applicable to reviews of observational studies [10] and they are *essential* for conclusions such as 'no evidence for a relation between X and Y’ to be drawn [3]. We hope to initiate reflections about scientific standards beyond the boundaries of disciplines and encourage researchers to tap the full potential of systematic reviews of observational studies in producing evidence relevant for policymakers [10]. We therefore call for high quality, rigorous systematic reviews on the effects of financial speculation on food prices/price volatility to address this important global health topic.

## Competing interests

KB received travel grants from medico international in 2009. The authors declare that they have no competing interests.

## Authors’ contributions

All authors contributed to the appraisal using AMSTAR and made substantial contributions to drafts and approved the final version of the manuscript. KB drafted the first version of the manuscript, revised subsequent versions, led the analysis of the appraisals together with SG and created the graph.

## Supplementary Material

Additional file 1 Supplementary webappendix. Click here for file
