# Supplementary material for: Relationship between financial speculation and food prices or price volatility: applying the principles of evidence-based medicine to current debates in Germany
Source: Global Health. 2013 Oct 16;9:44. doi: 10.1186/1744-8603-9-44 (PMC3819662; doi:10.1186/1744-8603-9-44)
Supplement: Additional file 1 — Supplementary webappendix. [file 1744-8603-9-44-S1.pdf]

Supplementary webappendix to:

Bozorgmehr K, Gabrysch S, Müller O, Neuhann F, Jordan I, Knipper M, Razum O. **Relationship between financial speculation and food prices or price volatility: Applying the principles of evidence-based medicine to current debates in Germany.** *Globalization and Health.*

Methodological aspects of the appraisal

General aspects

The appraisal team consisted of seven scientists with a background in public health, medicine and/or nutrition. The initial appraisals of all authors fed into the final analysis without any 'post-hoc' revisions based on face-to-face or tele-communication, which is in line with the usual AMSTAR procedures. Where indicators left space for interpretation, the members of the appraisal team provided information about the reasoning behind their choice of respective response options where necessary. All initial assessments were sent electronically to the first author (KB), who transferred the ratings into a spreadsheet for further analysis in STATA ® 12.1. and SPSS 16.0. Where members of the appraisal team made ambiguous judgements on an AMSTAR indicator (e.g. by ticking 'yes' and 'can't answer', because some essential aspects were reported but others not), the lead author (KB) chose the judgement in favour of Will et al. (though this was the case only once).

Application of AMSTAR indicators

While some indicators were more or less self-explanatory (e.g. AMSTAR2-6) and easily transferable to the review in question, others left space for interpretation. Therefore, we provide a brief narrative account on the rationale behind some of the ratings.

For example the negative ratings in AMSTAR7 (Figure 1, main text) were not given to the review due to a lack of 'randomized, double-blind, placebo controlled studies' (as listed in the notes of AMSTAR).

These criteria are neither applicable to most economic studies nor to observational studies in the field of medicine or public health. The negative rating was due to the general absence of any kind of 'a priori' method that indicated (transparently and in a replicable manner) how the quality of the included studies was assessed. The 'ad-hoc' assessment of the quality of included studies in Will et al. is prone to bias. This is, in our view, reflected in the unbalanced discussion of strengths and weaknesses of included studies, giving more weight to studies that show no effects, while discrediting the findings of those studies which provided evidence for an effect.
Consequently, AMSTAR8 was rated negatively as well, since this indicator (according to the AMSTAR online notes) should not score 'yes' in absence of any transparent method for quality assessment (i.e. if AMSTAR7 scored 'no', AMSTAR8 should not score 'yes'). An exception was made by one reviewer, who allowed AMSTAR8 to be rated 'yes' because of potentially different standards of assessing and weighing 'quality' of economic studies.

The largest discrepancy in ratings was found for AMSTAR9 (see Figure 1, main text). This indicator was rated 'not applicable' by the majority of reviewers since they felt it goes beyond their expertise to judge whether or not the study findings could have been pooled. A smaller proportion rated this item negatively, which was mainly due to the absence of a reference to heterogeneity in included studies (see the online AMSTAR note: "*Indicate "yes" if they mention or describe heterogeneity, i.e., if they explain that they cannot pool because of heterogeneity/variability.."*). Due to the controversial judgements we excluded AMSTAR9 from the analysis (see main text).

Finally, AMSTAR10, which refers to the possibility of publication bias received unanimously negative ratings. These ratings were due to the absence of any kind of reference to the possibility of publication or selection bias (especially given the narrow focus of the review on publications published in a period of two years only).

Potential sources of discrepancies in ratings

Due to the fact that all initial assessments fed into the review without any post-hoc 'corrections', it is principally possible that false positive or false negative judgements affected our results.

For example, one reviewer rated AMSTAR4 with 'no', although grey literature was indeed an inclusion criteria in the review in question. Further discrepancies might have occurred due to the fact, that some reviewers of the team used a paper-based version of AMSTAR while others additionally used the online AMSTAR checklist (http://amstar.ca/Amstar_Checklist.php), which provides some additional explanatory notes on each indicator above those provided in the paper-based version.

This might explain, for example, the discrepancies in AMSTAR1 (see Figure 1, main text): the online checklist refers to an 'a priori' design relatively strictly as a study that refers to a protocol, ethics approval, or pre-determined/a priori published research objective, while the paper-based version only demands that the research question and inclusion criteria should be established before the conduct of the review, which leaves more space for interpretation.

However, as Figure 1 and the high ICC suggest (see main text), such minor discrepancies did not considerably affect the overall judgement on the quality of the review.
